# Severe inbreeding, increased mutation load and gene loss-of-function in the critically endangered Devils Hole pupfish

**DOI:** 10.1098/rspb.2022.1561

**Published:** 2022-11-09

**Authors:** David Tian, Austin H. Patton, Bruce J. Turner, Christopher H. Martin

**Affiliations:** ^1^ Department of Integrative Biology, University of California, Berkeley, CA 94720, USA; ^2^ Museum of Vertebrate Zoology, University of California, Berkeley, CA 94720, USA; ^3^ Department of Biological Sciences, Virginia Tech, Blacksburg, VA 24061, USA

**Keywords:** inbreeding, runs of homozygosity, mutation load, Devils Hole pupfish

## Abstract

Small populations with limited range are often threatened by inbreeding and reduced genetic diversity, which can reduce fitness and exacerbate population decline. One of the most extreme natural examples is the Devils Hole pupfish (*Cyprinodon diabolis*), an iconic and critically endangered species with the smallest known range of any vertebrate. This species has experienced severe declines in population size over the last 30 years and suffered major bottlenecks in 2007 and 2013, when the population shrunk to 38 and 35 individuals, respectively. Here, we analysed 30 resequenced genomes of desert pupfishes from Death Valley, Ash Meadows and surrounding areas to examine the genomic consequences of small population size. We found extremely high levels of inbreeding (*F*_ROH_ = 0.34–0.81) and an increased amount of potentially deleterious genetic variation in the Devils Hole pupfish as compared to other species, including unique, fixed loss-of-function alleles and deletions in genes associated with sperm motility and hypoxia. Additionally, we successfully resequenced a formalin-fixed museum specimen from 1980 and found that the population was already highly inbred prior to recent known bottlenecks. We thus document severe inbreeding and increased mutation load in the Devils Hole pupfish and identify candidate deleterious variants to inform management of this conservation icon.

## 1. Introduction

Due to declining population sizes and increasing isolation of many species from anthropogenic habitat fragmentation and climate change, understanding the extent and nature of genetic threats in small populations is essential for predicting and increasing population persistence and resiliency [1]. Small and isolated populations often suffer from inbreeding depression, the reduction in fitness caused by increased homozygosity of deleterious recessive alleles or overdominant loci that occurs when closely related individuals breed together [2,3]. Prolonged population decline can result in increased long-term extinction risk due to stochastic demographic events [4], reduced genetic variation for adaptation [5], and decreased efficacy of purifying selection to overpower drift and purge deleterious variants [6]. This reduced efficacy of purifying selection leads deleterious mutations to accumulate more readily in small populations. The burden of accumulated deleterious mutations is known as the mutation load, and can reduce individual fitness [7,8].

Many examples of reduced genetic diversity and inbreeding depression have been documented in the wild [9,10], including Florida panthers (*Puma concolor*) [11], Isle Royale wolves (*Canis lupus*) [12] and mountain gorillas (*Gorilla beringei*) [13]. Severe inbreeding in natural populations is increasingly being documented across a wide range of temporal scales, from ancient bottlenecks [14,15] to recent timescales [12]. Recently, historical museum specimens have been successfully leveraged to investigate temporal changes in inbreeding and genetic diversity, highlighting their utility for investigating the historical dynamics of inbreeding and mutation load in imperilled populations [16,17].

Traditionally, population health and extinction risk was assessed using putatively neutral molecular markers and pedigrees owing to positive correlations between genome-wide heterozygosity and fitness [18]. Maintaining genome-wide genetic variation is crucial to preserving adaptive potential and preventing inbreeding depression in populations of conservation interest [19] because populations with higher levels of genetic diversity tend to have higher mean fitness and reduced extinction risk [20]. However, several recent studies have suggested that summary statistics of genetic variation do not necessarily accurately reflect population size or extinction risk and that we should also use whole-genome sampling to assess functional genetic diversity and genetic load [17,21]. Genomes enable more detailed measurements of individual inbreeding depression and its genetic basis relative to pedigree-based approaches [22]. Thus, a comprehensive understanding of the evolutionary dynamics of small populations in the wild from a genomic perspective is key to understanding the fate of endangered populations and to inform conservation management.

**Figure 1.**
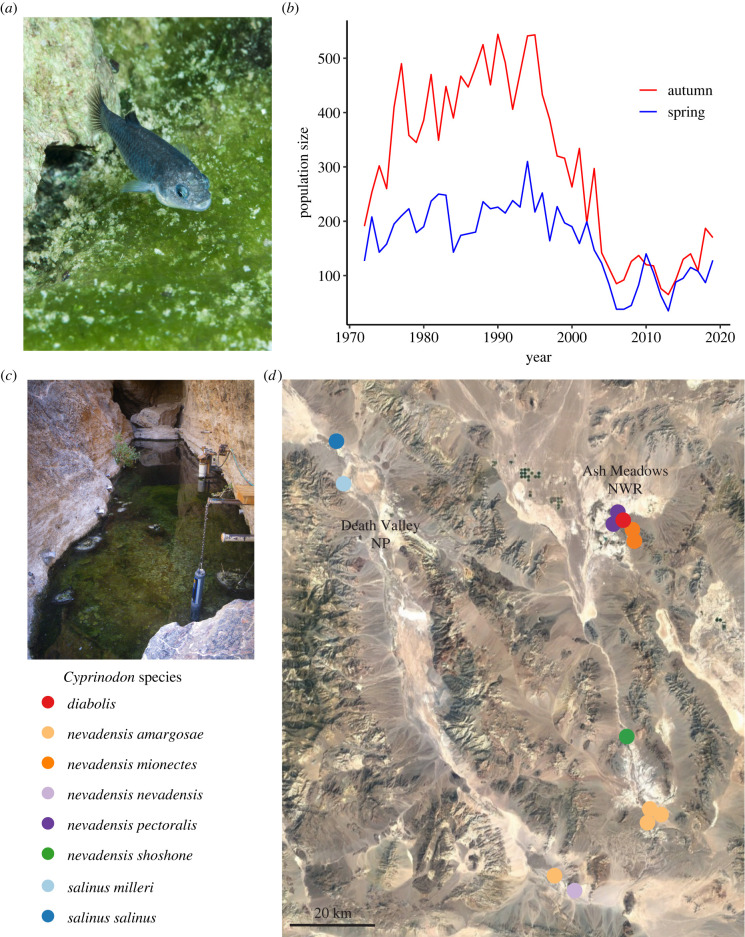
Sampling locations and recent *C. diabolis* population decline. (*a*) Photo of *C. diabolis* by Olin Feuerbacher. (*b*) Biannual population census counts of *C. diabolis* over time. Bottlenecks in 2007 and 2013 reached 38 and 35 individuals, respectively. (*c*) Photo of Devils Hole by USFWS. (*d*) Map of Death Valley NP and Ash Meadows NWR sampling locations. (Online version in colour.)

Here, we leverage the unique evolutionary and demographic history of the iconic Devils Hole pupfish (*Cyprinodon diabolis*: figure 1*a*) to investigate how isolation and recent population decline have shaped inbreeding and mutation load. Death Valley pupfishes evolved from a common ancestor thousands of years ago when the climate was milder and the region was connected by large inland seas. Populations are now relatively isolated in small desert spring systems and several species are now considered critically endangered [23]. Devils Hole contains the most extreme conditions, including a nearly constant temperature of 34°C [24], absence of direct sunlight during the winter [25] which sharply limits primary production and nutrient availability [26], and dissolved oxygen levels near lethal limits for most fishes (2–3 ppm) [25,27]. Surrounding pupfishes in neighbouring springs occupy less hypoxic environments, such as *Cyprinodon nevadensis mionectes* in Jackrabbit Spring, due to greater water movement [28].

*Cyprinodon diabolis* exists at one of the lowest long-term population sizes of any desert pupfish*.* The population has steadily declined since the late 1990s before reaching lows of 38 and 35 individuals in the spring of 2007 and 2013, respectively (figure 1*b*). *Cyprinodon diabolis* is restricted to the upper 30 m of Devils Hole, a 3.5 x 22 m water-filled cavern widely believed to be the smallest range of any vertebrate [29] (figure 1*c*). Population viability analyses in 2014 suggested that the median time to extinction was 26 years [30]. Although this species was previously believed to be isolated in Devils Hole for 10–20 ka (Miller [31]), more recent genome-wide estimates indicate that Devils Hole may have most recently experienced substantial admixture approximately 1–2 ka and that gene flow among these desert oases is surprisingly common [23,32,33].

The continued persistence of *C. diabolis* in the hottest desert on earth in one of the most inhospitable habitats for fishes is extraordinary. Despite its status as one of the world's most endangered species, genetic analyses have so far been limited to delineating phylogenetic relationships, assessing population structure, and measuring genetic diversity with reduced-representation genetic markers [23]. Here, we resequenced whole genomes of *C. diabolis* and several closely related *Cyprinodon* desert pupfishes to investigate how isolation and small population size influence inbreeding and mutation load on a genome-wide scale in this conservation icon.

## 2. Results

### (a) Geography and population structure

**Figure 2.**
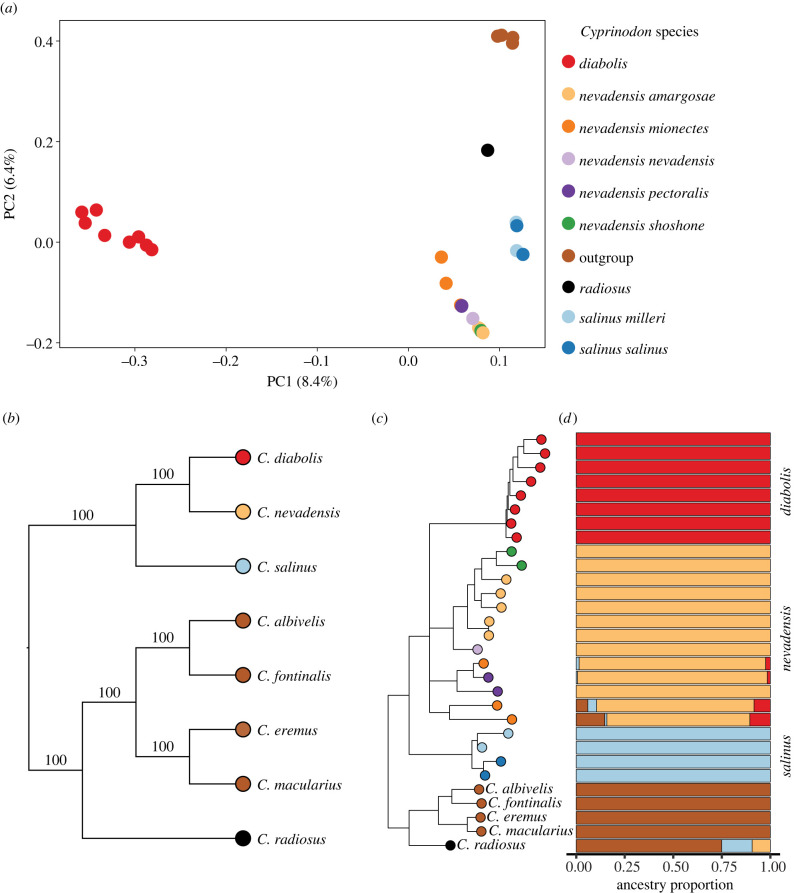
Population structure and evolutionary relationships among desert pupfishes. (*a*) Principal component analysis of desert pupfishes revealing substantial population structure among species and populations. (*b*) Species tree estimated by SVDquartets; nonparametric bootstrap support values indicate the percentage of bootstrap replicates supporting monophyly for each clade. (*c*) Maximum-likelihood tree of all 30 samples estimated by IQ-TREE. Two internal branches were collapsed due to low support (ultrafast bootstrap < 95%, SH-aLRT < 80%); all other branch support was unequivocal (UFboot = 100%, SH-aLRT = 100%). (*d*) Ancestry proportions across individuals in Death Valley NP, Ash Meadows NWR and outgroup desert pupfishes (*C. albivelis*, *C. eremus*, *C. fontinalis*, *C. macularius*, *C. radiosus*) estimated from a LD-pruned SNP dataset in ADMIXTURE with *k* = 4. Colours in PCA, trees and ADMIXTURE designate individuals from different species or populations and correspond to the shared figure legend in (*a*). (Online version in colour.)

We sequenced 30 individuals (8 *C. diabolis*, 13 *C. nevadensis*, 4 *C. salinus*, and one individual each of *C. albivelis*, *C. eremus*, *C. fontinalis*, *C. macularius* and *C. radiosus*) for our analyses (figure 1*d*, electronic supplementary material, tables S1 and S2). After filtering for quality genotypes and exclusion of problematic samples, we retained a total of 6 295 414 SNPs with a mean coverage of 12×. We investigated genome-wide population genetic differentiation among Death Valley desert pupfishes using principal component analysis, corroborating previous results [23] (figure 2*a*). Devils Hole pupfish were substantially divergent from the most closely related neighbouring desert pupfish species for genome-wide mean *F*_st_ estimates (*C. diabolis* versus *C. nevadensis* = 0.34). Inference of evolutionary relationships among these populations using genome-wide SNP data under the multi-species coalescent using SVDquartets [34,35] and concatenated SNP data across all individuals using IQ-TREE v.1.6.12 [36] confirmed previous findings; *C. diabolis* is sister to *C. nevadensis* and *C. salinus* is sister to both [23]. Our expanded sampling of outgroups also confirmed that the Death Valley clade is most closely related to the geographically proximate Owens pupfish (*C. radiosus*) [37]. ADMIXTURE analyses support *C. diabolis*, *C. nevadensis* and *C. salinus* as distinct populations. Interestingly, this analysis infers apparent admixture within *C. nevadensis* from neighbouring populations as more distantly related desert pupfishes (figure 2*d*); this complex history of admixture may help to explain the lack of phylogenetic resolution within this group.

### (b) Severe inbreeding in Devils Hole pupfish

**Figure 3.**
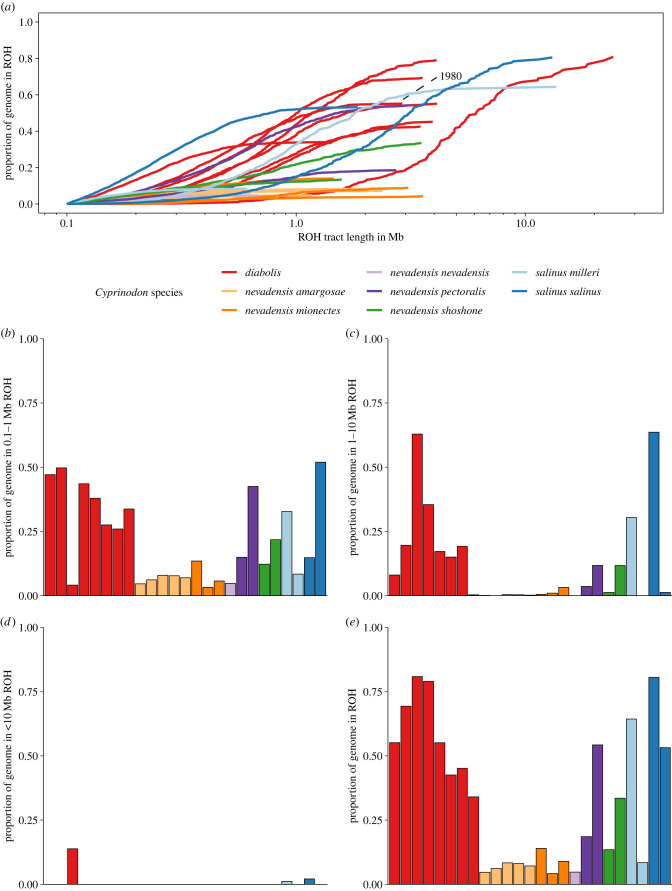
Extreme inbreeding in the Devils Hole pupfish. (*a*) *F*_ROH_ measured as the cumulative fraction of the genome made up of runs of homozygosity (ROHs) at least 100 kb long. (*b*) Proportion of the genome in ROHs of 0.1–1 Mb for each individual. (*c*) Proportion of the genome in ROHs of 1–10 Mb for each individual. (*d*) Proportion of the genome in ROHs greater than 10 Mb for each individual. (*e*) Total proportion of the genome within an ROH greater than 100 kb. (Online version in colour.)

Inbreeding can be identified and quantified through runs-of-homozygosity (ROHs), which are long contiguous tracts of identical haplotypes inherited from a common ancestor [22]. We calculated *F*_ROH_, an accurate measure of inbreeding, as the summed lengths of ROHs greater than 100 kb divided by the total genome size. We found that *C. diabolis* was highly inbred (mean *F*_ROH_ = 0.58), significantly exceeding the degree of inbreeding observed in *C. nevadensis* (mean *F*_ROH_ = 0.14: Tukey's HSD *p* = 8.38 × 10^−5^: figure 3). By contrast, ROHs made up less than 10% of the genome in the relatively undisturbed natural spring populations of *C. nevadensis amargosae* and *C. nevadensis nevadensis*, whereas *C. nevadensis shoshone* and *C. nevadensis pectoralis* tended to have a higher *F*_ROH_ than other *C. nevadensis* species (figure 3). These findings are consistent with small census population sizes and intensive management histories as *C. nevadensis shoshone* has experienced extirpation and captive breeding prior to reintroduction in the 1990s and the habitat of *C. nevadensis pectoralis* has undergone extensive habitat modification [38–40].

We found that the degree of inbreeding in Devils Hole has remained high, from 1980 (*F*_ROH_ = 0.55; *n* = 1) to near-present day (2008–2012: mean *F*_ROH_ = 0.58). This suggests that *C. diabolis* was already highly inbred prior to the population decline in the mid-1990s and severe bottlenecks in 2007 and 2013, during which the population size plummeted to 38 and 35 fish, respectively [30] (figure 1*b*).

Shorter ROHs indicate mating between distant relatives or longer-scale historical processes, whereas longer ROHs are indicative of recent inbreeding due to reduced opportunity for recombination [41]. We exploited this signature to estimate the mean number of generations back to the common ancestor of these homologous sequences using the length of ROHs and an assumed recombination rate. Populations with similar high levels of inbreeding can have ROHs that nearly span entire chromosomes [42]. Surprisingly, given the recent severe bottlenecks, we did not find many long ROHs (greater than 10 Mb) in our *C. diabolis* samples, barring a single exception (figure 3*d*). Instead, much of the cumulative inbreeding is made up of ROHs that are 0.1–1 Mb long (figure 3*b*), which corresponds to shared parental ancestry from 11 to 109 generations previous. Our results illustrate that extreme isolation and prolonged small population size have driven *C. diabolis* to become highly inbred and that much of the inbreeding occurred prior to recent bottlenecks over the course of the twentieth century.

### (c) Higher mutation load in Devils Hole pupfish

**Figure 4.**
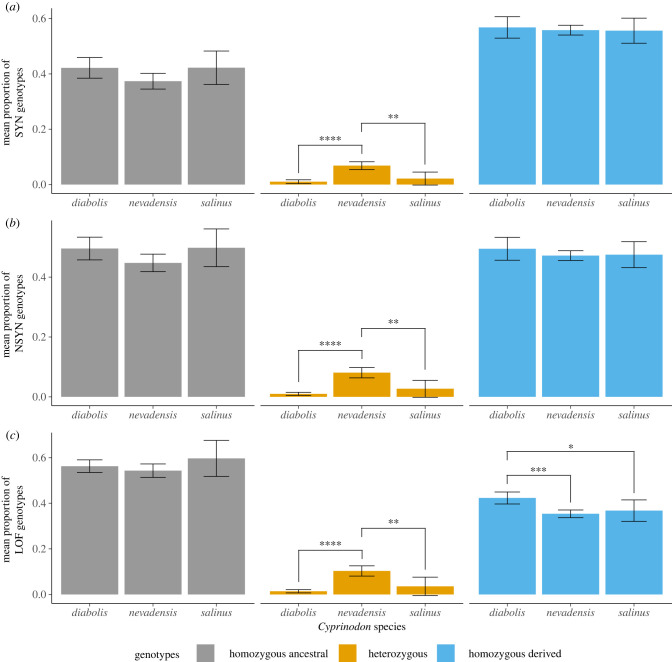
*Cyprinodon diabolis* suffers from uniquely high mutation load. The mean proportion of homozygous ancestral, heterozygous and homozygous-derived genotypes in *C. diabolis*, *C. nevadensis* and *C. salinus* with respect to the *C. brontotheroides* genome at segregating sites across all populations in coding regions. Variants were categorized into mutation classes as synonymous (SYN), non-synonymous (NSYN) or loss-of-function (LOF) with Snpeff. Loss-of-function mutations are defined as those that encode a premature stop codon. Error bars correspond to the 95% confidence interval, spanning the mean ± two standard errors. (Online version in colour.)

Small, inbred populations are expected to have higher frequencies and homozygosity of deleterious alleles. However, once deleterious recessive alleles are unmasked, purifying selection can also purge portions of the mutation load [43]. To assess how severe inbreeding in Devils Hole pupfish has affected mutation load, we calculated the relative proportions of homozygous ancestral, heterozygous and homozygous-derived genotypes across synonymous (SYN), non-synonymous (NSYN) and loss-of-function (LOF) mutations (figure 4; electronic supplementary material, figure S1). The number of homozygous-derived genotypes for LOF variants quantifies load under a recessive model [8,44]. Because deleterious alleles tend to be recessive [45,46], LOF homozygous-derived alleles are more likely to have a phenotypic effect that leads to a reduction in fitness.

*Cyprinodon diabolis* had significantly lower proportions of heterozygous genotypes than *C. nevadensis* across all variant types (SYN: *p* = 1.42 × 10^−5^, NSYN: *p* = 1 × 10^−5^, LOF: *p* = 1.87 × 10^−5^, Tukey's HSD tests), consistent with our findings of higher inbreeding in this species. *Cyprinodon diabolis* also had significantly higher proportions of homozygous-derived LOF genotypes compared to both *C. nevadensis* (*p* = 6.61 × 10^−4^) and *C. salinus* (*p* = 0.044). Similarly, *C. salinus* also had significantly lower proportions of heterozygous genotypes than pooled *C. nevadensis* samples across all variant types (SYN: *p* = 2.74 × 10^−3^, NSYN: *p* = 3.71 × 10^−3^, LOF: *p* = 5.36 × 10^−3^, Tukey's HSD tests), likely reflecting the larger population sizes of *C. nevadensis* populations. There were no significant differences in the proportion of homozygous-derived genotypes for SYN and NSYN mutations among the three species.

We also assessed whether there were allele frequency differences among the three species across a comparable set of LOF alleles and found that there were no significant differences among the three species (ANOVA *p* = 0.134), although *C. diabolis* tended to have a higher mean frequency of LOF alleles at 0.63, compared to *C. nevadensis* = 0.54 and *C. salinus* = 0.49. Finally, there was no enrichment of LOF variants in ROHs for most individuals except for three *C. diabolis* individuals that had significantly greater proportions of LOF variants in ROHs than their respective *F*_ROH_ values (DHP54903: *p* = 1.92 × 10^−3^, DHP54913: *p* = 4.29 × 10^−3^, DHP54918: *p* = 4.71 × 10^−2^). One *C. nevadensis amargosae* individual had a significantly lower proportion of LOF variants in ROHs (CNevAma: *p* = 2.01 × 10^−2^).

### (d) Fixed variants unique to Devils Hole

Devils Hole pupfish clearly harbour a homozygous LOF mutation load greater than neighbouring desert pupfish. Thus, we focused on genetic variants most likely to be deleterious to help inform future management of this species; specifically, homozygous-derived LOF variants and deletions unique to *C. diabolis*, which are expected to reduce fitness by disrupting gene function.

We identified 11 predicted LOF SNPs in the form of premature stop codons, including several within genes that may affect fecundity or resistance to disease and stress (electronic supplementary material, table S3). These include *cfap43*, a protein involved in the structure and function of the sperm flagellum axoneme that has been implicated in male infertility [47]. Similarly, reduced sperm motility and abnormal sperm morphology were observed in Florida panthers [11] and lions [48] due to inbreeding.

We also identified 94 deletions unique to *C. diabolis* and focused on the 15 deletions within 2 kb of any annotated genes. Surprisingly, five of the fifteen deletions were involved in cellular responses to hypoxia (electronic supplementary material, table S4), including shifts to anaerobic metabolism, erythropoiesis, degradation of misfolded proteins and regulation of nutrient use and cell fate [49]. These hypoxia-related deletions included an 81 bp deletion in the promoter of *redd1*. During hypoxia, *redd1* inhibits mTORC1 [50,51] through the TSC1/TSC2 tumour suppressor complex to conserve energy and prevent the accumulation of misfolded proteins. Upregulation of a homologue of *redd1* (*ddit4L*/*redd2*) has been implicated in adaptation to hypoxia in shortfin mollies (*Poecilia mexicana*) [52] and high-altitude deer mice (*Peromyscus maniculatus*) (N. R. Rochette 2021, personal communication). We also found deletions in *apeh*, an enzyme that destroys oxidatively damaged proteins [53]; t*rim39*, a tripartite motif involved in erythropoiesis [54] and *slc25a42*, a mitochondrial transporter of coenzyme A [55] associated with mitochondrial myopathy and lactic acidosis in humans [56].

## 3. Discussion

We documented extensive inbreeding, gene loss and mutation load in the critically endangered Devils Hole pupfish and identified a set of candidate deleterious genetic variants that can potentially inform future conservation management. We show that *C. diabolis* is significantly more inbred than most neighbouring desert pupfish populations*. Cyprinodon diabolis* was not significantly more inbred than *C. salinus* although our small sample size and high variability make it difficult to infer accurate levels of inbreeding in *C. salinus*. High levels of inbreeding are associated with elevated extinction risk [57,58] and the inbreeding in *C. diabolis* is equal to or more severe than levels reported so far in other isolated natural populations such as Isle Royale wolves [12], mountain gorillas [59] and Indian tigers [60]. Although we were unable to directly measure fitness, the increased inbreeding in *C. diabolis* likely results in a substantial reduction in fitness. Previous studies have suggested that increases in *F*_ROH_ have strong negative effects on fitness. For instance, in Soay sheep an increase in *F*_ROH_ by 10% was correlated with a 60% decline in fitness [61], whereas in helmeted honeyeaters a 9% increase in homozygosity was associated with a reduced lifetime reproductive success of 87–90% [62]. Surprisingly, *C. salinus* also displayed high levels of inbreeding although increased sampling is necessary to obtain reliable estimates of inbreeding.

By successfully sequencing a formalin-fixed historical museum specimen to 10× coverage (out of 8 total attempts) we discovered that inbreeding was already extensive by 1980, suggesting that *C. diabolis* may have an extended history of repeated population bottlenecks. Indeed, the distribution of ROH sizes suggests that a large proportion of homozygous tracts were due to inbreeding that occurred many generations prior to the recent population decline. Furthermore, *C. diabolis* harbours a significantly greater mutation load than either *C. nevadensis* or *C. salinus* (figure 4).

Our mutation load results support previous hypotheses that *C. diabolis* harbours a high mutation load due to its relative isolation and small population size [29,63]. Additionally, they are consistent with the observation of rapid allele frequency increases of *C. nevadensis* alleles in a *C. diabolis* refuge population after the accidental introduction of a few individuals [64]. Although recent studies have suggested that small, bottlenecked populations may harbour a lower mutation load due to purging [13,65,66], populations that have experienced recent severe population bottlenecks are likely to maintain a high load, because deleterious variants may reach fixation before purifying selection removes them [67]. Although our measure of mutation load does not directly measure fitness, there is empirical support that LOF mutations are on average deleterious [68].

### (a) Degradation of hypoxia and reproductive pathways in *Cyprinodon diabolis*

We found deleterious variants associated with reproduction and hypoxia genes. For example, we found a fixed LOF variant unique to *C. diabolis* in *cfap43*, a gene associated with sperm morphology and function. Deletions have thus far been rarely studied or quantified in conservation genetics. However, analysis of woolly mammoth genomes from different time points found that a sample dated closer to the time of extinction had accumulated more homozygous deletions than earlier ones [15], suggesting that deletions may be an understudied genetic threat to endangered populations [17].

We found numerous deletions that were unique to the Devils Hole pupfish. Of these, five were associated with hypoxia, a known environmental stressor in the system, suggesting that *C. diabolis* could be poorly equipped to physiologically deal with the stressful hypoxic environment in Devils Hole. Indeed, previous studies have noted that *C. diabolis* has low fecundity [24,69], low egg viability and juvenile survivorship [29] and lays more eggs at lower temperatures (28°C) compared to the higher constant temperature of 33°C in Devils Hole [70]. At present, we cannot rule out the possibility that some of these fixed variants are potentially the result of local adaptation; distinguishing between selection and genetic drift in small populations is extremely difficult because both processes leave similar signatures in allele frequencies [71]. One possibility is that these variants are adaptive in the unique selective environment of Devils Hole and were swept to fixation during initial colonization. Moving forward, functional genetic and eco-physiological tests will be key to understanding the full impacts of these variants. Our results highlight the importance of investigating deletions and structural variants to better understand unique genetic variation in endangered populations.

### (b) Did severe inbreeding cause the recent population decline?

The Devils Hole pupfish population began to decline in the 1990s from historical population sizes of 200–500 to less than 100 individuals for reasons still unknown (although in the most recent census the population has rebounded to 175 fish). Ecological hypotheses include declines in ostracod prey for juvenile pupfish [72,73] and changes in the dominant primary producers from *Spirogyra* algae to diatoms and cyanobacterial mats [74]. Furthermore, climate change may be shortening the seasonal period of optimal hatching conditions on the shallow shelf where *C. diabolis* spawns [75]. The stressful environment of present day Devils Hole may thus have exacerbated inbreeding depression, which typically increases under environmental stress [76,77].

Alternatively, previous studies speculated that the population has a high genetic load which led to ‘mutational meltdown’ based on the discovery of a dramatic shift towards predominantly *C. nevadensis* ancestry following introduction of a few *C. nevadensis* into a *C. diabolis* refuge population [64]. We estimated the effective population size of *C. diabolis* based on the harmonic mean of biannual census data ($N_{e}^{\mathrm{Spring}}=122$, $N_{e}^{\mathrm{Autumn}}=209$) and found that this species has an effective population size far below the suggested minimum for the maintenance of sufficient genetic variation for adaptive capacity [78,79].

#### (i) Past and future management actions

Initial management of the species involved various attempts to create wild refuge populations in the 1950s–1970s prior to the landmark 1976 US Supreme Court decision mandating a minimum water level, which halted habitat reduction and population decline caused by groundwater pumping [80]. Subsequent attempts to maintain refuge populations of *C. diabolis* in aquaria and semi-natural outdoor pools largely failed to sustain captive breeding colonies for long periods of time [29,64]. In response to the severe population bottlenecks in the early 2000s, Ash Meadows Fish Conservation Facility was constructed in 2012 to establish a refuge population that more closely mimicked the Devils Hole habitat and the refuge colony now outnumbers the wild population. Although we relied on degraded tissue samples collected from dead pupfish retrieved from Devils Hole for genetic analyses in this study, increasing population numbers may soon allow for tissue samples or functional genetic approaches in refuge live specimens.

## 4. Conclusion

Our study adds to a growing number of studies that measure inbreeding and mutation load in wild populations, with a novel focus on gene deletions in a wild highly inbred population [60,66]. The demographic history and genome-wide measures of high inbreeding and mutation load in the Devils Hole pupfish suggest that the population remains in danger. While successfully sequencing formalin-fixed samples remains difficult, we were able to do so for a single *C. diabolis* specimen from 1980 and found that the population was likely highly inbred prior to the recent bottleneck. The increasing availability of genomic sampling spanning multiple time points for endangered species such as the Devils Hole pupfish will better inform our understanding of inbreeding, mutation load, and specific putatively deleterious variants in this system, ultimately allowing for conservation management to monitor potentially harmful variation in wild and captive populations over time. Finally, we caution that targeted genetic management of deleterious variants should not be undertaken until candidates have been verified to have fitness consequences.

## 5. Methods

### (a) Samples and sequencing

We sequenced 44 whole genomes including *C. diabolis* (*n* = 23), *C. nevadensis* (*n* = 13) and *C. salinus* (*n* = 4) spanning multiple independent springs, along with closely related desert pupfish species from California, Arizona and Mexico (electronic supplementary material, table S1). Of the 23 *C. diabolis* genomes, eight were from historical museum samples, spanning 1937–1980, while the rest were non-destructively sampled between 2008 and 2012. Given the critically endangered status of *C. diabolis*, NPS and USFWS staff collected and preserved dead specimens found during routine checks during this period. All other species were collected in the early 1990s [81]. Samples were sequenced on 150 PE runs using an Illumina Novaseq. Historical and several degraded *C. diabolis* samples were prepared using Swift 2S Turbo library kits (Swift Biosciences). All sample metadata are reported in electronic supplementary material, table S1. Fourteen samples were excluded from downstream analyses due to a low percentage of reads mapping to the reference genome (less than 70%), improperly paired reads (less than 70%), or significant amounts of missing data per individual following SNP calling (greater than 80%), presumably due to degradation (electronic supplementary material, table S2: see electronic supplementary material for additional details). Following the filtering described above to identify high-quality samples, we retained for all downstream analyses the following 30 samples: 8 *C. diabolis*, 13 *C. nevadensis*, 4 *C. salinus*, and five closely related desert pupfish species (*C. albivelis*, *C. eremus*, *C. fontinalis*, *C. macularius*, *C. radiosus*) (figure 2).

### (b) Alignment and filtering

Raw reads were mapped from all 44 individuals to the *Cyprinodon brontotheroides* reference genome (UCB_Cbro_1.0; total sequence length = 1 162 855 435 bp; scaffold N50 = 32 Mbp) [82] with bwa-mem (v.0.7.12) [83]. Duplicate reads were identified using MarkDuplicates and BAM indices were created using BuildBamIndex in the Picard software package (http://picard.sourceforge.net; v.2.0.1). We followed the best practices guide recommended in the Genome Analysis Toolkit (v.3.5) [84] to call and refine our single nucleotide polymorphism (SNP) variant dataset using the program HaplotypeCaller. SNPs were filtered based on the recommended hard filter criteria (QD < 2.0; FS > 60; MQ < 40; MQRankSum < −12.5; ReadPosRankSum < −8) because we lacked high-quality known variants. See electronic supplementary material for additional details. Our final dataset contained 6 295 414 SNPs.

### (c) Population structure

We pruned SNPs in strong linkage disequilibrium using the LD pruning function (–indep-pairwise 50 5 0.5) in PLINK (v.1.9) [85] leading to the retention of 1 653 597 variants. We then characterized population structure with two approaches. First, we used PLINK to conduct principal component analysis. Second, we used ADMIXTURE (v.1.3.0) [86] to assign individuals to variable numbers of population clusters (*K* = 1–20). We used the subset parameter in PLINK to randomly select 100 000 SNPs for analysis. We calculated genome-wide *F*_st_ between *C. diabolis* and *C. nevadensis*, based on the 6.3 million SNP dataset using the weir-fst-pop function in vcftools (v.0.1.15) [87].

### (d) Phylogenetic inference

Phylogenies were inferred at the individual and species-level from 1 653 597 LD-pruned SNPs using IQ-Tree (v.1.6.12) [36] and SVDquartets [34,35]. Full details are provided in the electronic supplementary material.

### (e) Population size

Census data for 1972–spring 2013 was acquired from Beissinger [30]. Data for subsequent counts were based on released NPS news reports. Variance effective population size was calculated as the harmonic mean of spring (*N_e_*^Spring^) and autumn (*N_e_*^Autumn^) population counts over time.

### (f) Runs of homozygosity

Runs of homozygosity (ROHs) were identified in our Death Valley samples from the full filtered set of 6 295 414 SNPs using the BCFtools/ROH command (v.1.14) [88], which uses a hidden Markov model to identify homozygous portions of the genome from genetic variation data. To calculate the cumulative fraction of the genome consisting of ROHs (*F*_ROH_) for each individual, we summed the lengths of identified ROHs with length greater than 100 kb and divided by the total size of the genome (1 162 855 435 bp). We also classified ROHs into various lengths of 0.1–1 Mb, 1–10 Mb, and greater than 10 Mb and calculated the proportion of the genome that these classes of ROHs comprised in a similar fashion. Although the amount of missing data was significantly associated with our measure of FROH across all samples (electronic supplementary material, figure S2), we found no significant association between FROH and depth of coverage or missing data for *C. diabolis* samples alone. Furthermore, we were still able to detect low FROH in two *C. nevadensis* samples with large amounts of missing data. The age of ROHs was estimated as *g* = 100/2 × ROH length (cM), where *g* is the number of generations to the most recent common ancestor [41]. We assumed a generation time of 1 year [29], and an average recombination of approximately 4.6 cM/Mb (genetic map length = 5330 cM [89]; *C. brontotheroides* genome size = 1.16 Gb [82]).

### (g) Measuring mutation load

We categorized variants in coding regions based on their putative effect on the amino acid sequence (i.e. LOF or NSYN) and whether the alleles were derived with respect to our reference (*C. brontotheroides*) genome using SnpEff [90]. LOF variants were conservatively defined as SNPs that resulted in a premature stop codon [65,91], which are expected to be less prone to misannotation [92]. Unique, putatively deleterious variants were defined as being: (i) homozygous derived, (ii) a gained stop codon and (iii) present in all of our *C. diabolis* samples for which genotypic information was available and absent in all other non-*C. diabolis* samples in our dataset. See electronic supplementary material for additional details.

We did not rely on direct counts of variants or derived alleles as they are significantly positively correlated with coverage (electronic supplementary material, figure S3) and missing data (electronic supplementary material, figure S4). Instead, we measured mutation load in terms of the proportions of SYN, NSYN and LOF genotypes that were homozygous ancestral, heterozygous or homozygous-derived across species (e.g. [91]). This transformation largely corrected for the confounding effect of coverage (electronic supplementary material, figure S5) and missing data (electronic supplementary material, figure S6) on our estimates of mutation load. We assessed whether mutation loads differed among the three species using an ANOVA in R and used Tukey's HSD tests in R to test for pairwise differences among species [93]. To compare allele frequencies of LOF alleles between our three species, we first identified 62 LOF variants for which there was genotype information for at least four individuals per species and for which the variant was present in at least two of the three species, to control for differences in sampling number among species. We then calculated species-specific allele frequencies for each of these 62 LOF variants. Differences in the average allele frequency of LOF alleles were assessed using an ANOVA in R [93]. We assessed whether LOF variants were enriched in ROHs for each individual by performing binomial tests where the number of successes was defined as the number of LOF variants in ROHs, the number of trials was defined as the total number of LOF variants, and the probability of success was defined as the individual's *F*_ROH_.

### (h) Unique deletions

We identified deletions that were unique to *C. diabolis* using DELLY (v.0.8.3) [94]. We only characterized deletions that were present in our five highest quality *C. diabolis* samples (DHP1980-5, DHP54903, DHP54913, DHP54917, DIAB54919), but absent in other species. Deletions that were exceptionally large and presumably artefacts were checked for accuracy in IGV and subsequently removed (*n* = 4). We focused primarily on deletions within 2 kb of an annotated gene. Deletions were confirmed to be unique to *C. diabolis* by aligning BAM files spanning the deletion and confirming their presence in *C. diabolis* and absence in non-*C. diabolis* samples. We analysed whether deletions spanned exons, introns or regulatory regions by BLASTing [95] deleted sequences against the C_variegatus-1.0 (GCF_000732505.1) assembly on Ensembl (release 102; [96]).

## Data Availability

All raw genomic data are archived at the National Center for Biotechnology Information BioProject Database (Accession: PRJNA887195). Genotypic data and population census data are available through the Dryad Digital Repository: https://doi.org/10.6078/D1ND9Q [97]. Scripts are available at https://github.com/tiandavid/Cyprindon_WGS_project/. The data are provided in electronic supplementary material [98].
